# Automated discretization of ‘transpiration restriction to increasing VPD’ features from outdoors high-throughput phenotyping data

**DOI:** 10.1186/s13007-020-00680-8

**Published:** 2020-10-16

**Authors:** Soumyashree Kar, Ryokei Tanaka, Lijalem Balcha Korbu, Jana Kholová, Hiroyoshi Iwata, Surya S. Durbha, J. Adinarayana, Vincent Vadez

**Affiliations:** 1 Centre of Studies in Resources Engineering, Indian Institute of Technology Bombay, Mumbai, India 400076; 2 Laboratory of Biometrics and Bioinformatics, University of Tokyo, Tokyo, Japan; 3 Debre Zeit Research Center, Ethiopian Institute of Agricultural Research (EIAR), Debre Zeit, Ethiopia; 4 International Crop Research Institute for Semi-Arid Tropics, Hyderabad, India 502319; 5 Institut de Recherche Pour Le Développement (IRD), Université de Montpellier—UMR DIADE, 911 Avenue Agropolis, BP 64501, 34394 Montpellier cedex 5, France

**Keywords:** High throughput phenotyping, Transpiration rate, Vapor pressure deficit, Time series, Machine learning, Feature selection, Unsupervised random-forest, Gini index, Neural network, Sensitivity analysis

## Abstract

**Background:**

Restricting transpiration under high vapor pressure deficit (VPD) is a promising water-saving trait for drought adaptation. However, it is often measured under controlled conditions and at very low throughput, unsuitable for breeding. A few high-throughput phenotyping (HTP) studies exist, and have considered only maximum transpiration rate in analyzing genotypic differences in this trait. Further, no study has precisely identified the VPD breakpoints where genotypes restrict transpiration under natural conditions. Therefore, outdoors HTP data (15 min frequency) of a chickpea population were used to automate the generation of smooth transpiration profiles, extract informative features of the transpiration response to VPD for optimal genotypic discretization, identify VPD breakpoints, and compare genotypes.

**Results:**

Fifteen biologically relevant features were extracted from the transpiration rate profiles derived from load cells data. Genotypes were clustered (C1, C2, C3) and 6 most important features (with heritability > 0.5) were selected using unsupervised Random Forest. All the wild relatives were found in C1, while C2 and C3 mostly comprised high TE and low TE lines, respectively. Assessment of the distinct p-value groups within each selected feature revealed highest genotypic variation for the feature representing transpiration response to high VPD condition. Sensitivity analysis on a multi-output neural network model (with R of 0.931, 0.944, 0.953 for C1, C2, C3, respectively) found C1 with the highest water saving ability, that restricted transpiration at relatively low VPD levels, 56% (i.e. 3.52 kPa) or 62% (i.e. 3.90 kPa), depending whether the influence of other environmental variables was minimum or maximum. Also, VPD appeared to have the most striking influence on the transpiration response independently of other environment variable, whereas light, temperature, and relative humidity alone had little/no effect.

**Conclusion:**

Through this study, we present a novel approach to identifying genotypes with drought-tolerance potential, which overcomes the challenges in HTP of the water-saving trait. The six selected features served as proxy phenotypes for reliable genotypic discretization. The wild chickpeas were found to limit water-loss faster than the water-profligate cultivated ones. Such an analytic approach can be directly used for prescriptive breeding applications, applied to other traits, and help expedite maximized information extraction from HTP data.

## Background

To reach maturity, crops under water stress must match water consumption to water availability [1]. For the majority of crops, and particularly those of the semi-arid tropics, this implies having to deal frequently with high Vapor Pressure Deficit (VPD) in the air, which creates a situation of an atmospheric water stress [2, 3]. In this regard, ‘the capacity to restrict or limit transpiration under high VPD’ is a promising trait that alleviates that stress, allows water saving, and increases yield through sustained growth under terminal drought conditions [2, 4]. Many researchers suggest the inclusion of this trait in the cultivars typically grown in water-limited environments [5, 6].

So far, the capacity to restrict transpiration under increasing VPD conditions has been mostly measured in growth chambers using constant light and increasing VPD levels [7–9]. The features used to compare genotypes were the slopes of the linear models representing the transpiration response, and possible VPD breakpoints where those slopes would change. Furthermore, in some cases VPD is increased by altering only the relative humidity percentage [10], although temperature is also known to influence the ability to control transpiration in response to VPD [11]. Such analyses not only suffer from a very low throughput, but the transpiration profiles obtained thereof only partially represent the natural conditions (since light or temperature remain constant during those experiments). Therefore, the theoretical representation of this trait (i.e. a limited maximum transpiration rate (TR) value in the high VPD hours of the day) described in a crop model [4] is also partially featured. Only few measurements have been done under natural conditions [12–16], with both temperature and relative humidity changing simultaneously over the course of the day. Those studies measured the expression of genotypic differences in the transpiration response to VPD only from the maximum transpiration values under high VPD conditions, and also had low throughput. No study has attempted to exhaustively identify additional features that could represent the transpiration profile differences under high VPD, at a high throughput level and precision, and in a systematic manner.

Existing High-Throughput Phenotyping (HTP)-based analysis of canopy-conductance mechanisms majorly employ daily estimates of evapotranspiration (ET) [17, 18]. However, under field conditions canopy-conductance patterns among plants are quite dynamic and diverse within a day due to simultaneous changes in environmental variables [13, 19]. Capturing that diversity requires measurement of transpiration at high frequencies [19]. Until recently, very few studies have measured transpiration at high frequencies (although under controlled conditions) viz. 3 min [20] and 10 min [15]. Therefore, in this paper, data from the HTP platform, LeasyScan (LS) [13] is used. The LS experimental set up has been expanded to 1488 load cells (LC), the gravimetric sensors that weigh each tray or sector every 15 min from which ET is calculated. Plant leaf area is simultaneously measured through 3-dimensional (3D) laser scanners, and allows the assessment of in-vivo canopy conductance traits. Thus, the LS platform enables HTP of crop ET over time.

However, data generated in such platforms is not only huge but also contain noise, that mostly occur due to occasional fluctuations in the millivolt signals produced by the load cells, which alter the load cell readings. Noise also prevails around irrigation or rain events that cause sharply increasing and decreasing trajectories in the profiles of sector weights [13, 20]. Therefore, conversion of raw load cell data into interpretable transpiration responses requires: (i) meticulous de-noising of the load cell readings considering the variations in ambient conditions; (ii) developing clean transpiration profiles from which features that distinguish genotypic differences in the transpiration response to VPD can be systematically extracted. The existing articles on HTP of such functional traits have not yet considered the importance of these aspects, and have used simple averaging of load cell readings to generate smooth transpiration profiles [15, 20]. This limits the information that can be gathered from these transpiration profiles, for instance how the environmental factors drive the changes in water loss patterns. Thus, there remains a gap in the availability of generic and efficient approaches to enable convenient handling of such voluminous HTP data, and maximizing information extraction.

Therefore, considering these challenges and gaps, a machine learning (ML) -based framework is presented that majorly employs two state-of-the-art ML algorithms, Random Forest (RF) and Neural Networks (NN) besides others. In this study, RF is employed for optimally clustering the genotypes and identifying the most significant transpiration rate (TR) features, while NN is used to model the relationships between TR of each cluster and the weather variables, at different levels of changing ambient conditions. RF is an ensemble ML algorithm that uses bagging (random subsets of samples; here genotypes) and feature randomness (random subsets of TR features, in this case) to train a multitude of uncorrelated trees, independently. The final model outcome is based on the decisions of the ensemble as a whole, and not as a single tree model. Thus, RF inherently handles the limitations of sensitivity and bias through bagging and feature randomness, respectively [21]. It is also one of the best-known feature selection algorithms [22, 23], besides being implemented for both supervised and unsupervised learning [23, 24]. NN, on the other hand is one of the best suited algorithms for efficient modeling of complex relationships between multiple responses and multiple predictors e.g. plant-environment interactions [25–27]. The network can be designed with several layers between the inputs and the output(s), where each layer (hidden) leverages some activation function to successively distill important patterns from its inputs and transmit to the next layer. In essence, by controlling the hidden layers and activation functions, any level of complexities or non-linearities can be precisely captured by the network. Finally, the user is offered with the most informative patterns of the data while also being abstracted from the details of signal transmissions among the neurons. Both RF and NN make no prior assumptions of the underlying structure of data, and are therefore preferred for modeling non-linear relations like plant-environment interactions [23, 28].

The specific objectives of the study are thus, to:

i. Seamlessly convert high-frequency load cell data into continuous TR profiles (without compromising the frequency of the original load cell time series),
ii. Extract and select potential data-driven TR features, that could best serve as proxy phenotypes for clustering genotypes based on the differences in their water-saving trait,
iii. Precisely quantify the breakpoint (i.e. the VPD levels at which maximum TR of each cluster starts limiting) through sensitivity analysis of the plant-environment interactions.

## Materials and methods

### Test-site and HTP data description

**Fig. 1:**
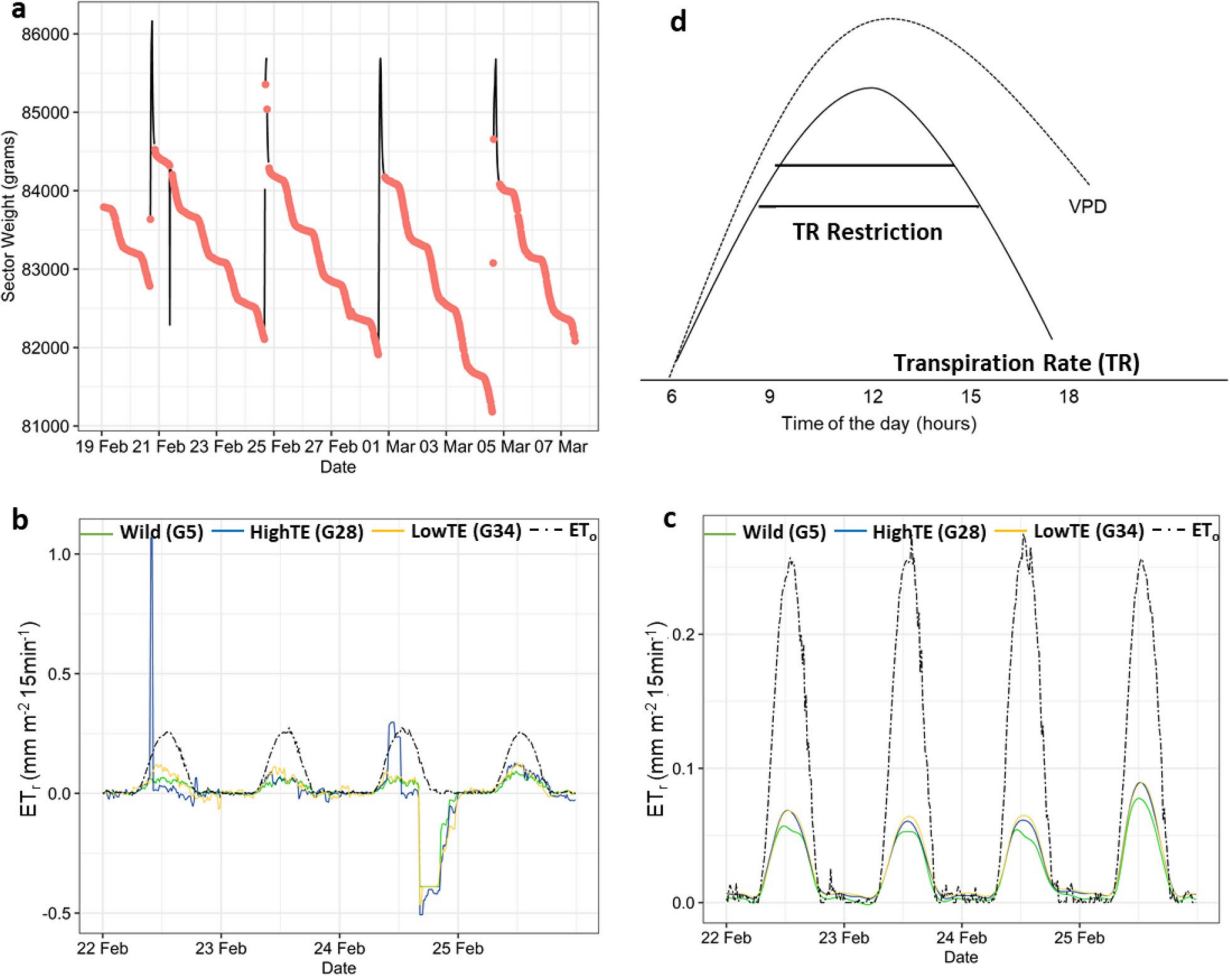
**a** An example of raw load cell data (black lines) and its time series after MODWT-based outlier removal (red points) shown for the duration 19.02—07.03.2017. The ETr of wild (G5), highTE (G28) and lowTE (G34) genotypes obtained **b** before and **c** after smoothing are overlaid with the reference ET (ET_0_) time series and shown for the duration 22—25.02.2017. **d** Theoretical representation of the water-saving trait that suggests restriction of maximum transpiration rate at high VPD conditions

The time series data set analyzed in this work comprised load cell weights collected every 15 min’ interval, from 20th February to 6th March 2017 (see Additional file 1: Table S1 for details on observed environmental conditions in that duration). The dataset represented the growth profile of 48 chickpea genotypes, measured in an Alpha-Lattice design with 4 replications, at the LS HTP platform, ICRISAT-Patancheru (17.5111° N, 78.2752° E). Plants were grown in large trays (40 × 60 × 30 cm, length–width-height) filled with vertisol collected from the ICRISAT farm, and containing 8 plants per tray. The trays in which plants were cultivated were adjacent to one another and were arranged in blocks of 12 × 2 trays (4.8 m × 1.3 m), so that the canopy of one tray eventually joined the canopy of the following tray and created a field-like canopy over the extent of each block. Planting was done on 6th February so that plants were at a vegetative stage for the time series data set considered here. Soil was fertilized with di-ammonium phosphate (DAP) at sowing at a rate of 6 g per tray. Trays were maintained fully irrigated throughout the experiment with a drip irrigation system, every 3–4 days back to field capacity, and received 4 irrigations in the timeframe of the analysis (see Fig. 1a). The 48 genotypes included 16 wild relatives of chickpea (i.e. the wild group) and 32 cultivated chickpea genotypes. Among the cultivated lines, 12 were cultivated checks, 10 genotypes were previously found to have high transpiration efficiency (TE) i.e. the highTE group, whereas the other 10 genotypes had low TE, the lowTE group (Vadez, personal communications). From these TE differences, those genotypes could be expected to have different patterns of transpiration under high VPD [1].

### Extraction of TR from load cells time series

#### Conversion of load cells observations to evapotranspiration time series

Load cells data collected from the platform were noisy at times, due to noise inherent to the electronic systems (low frequencies, or amplitude, over the entire duration). Other fluctuations occurred during the days of irrigation with anomalous spikes in sector weights caused by watering and drainage of excess water (i.e. high frequencies, corresponding to large changes in the patterns of the load cell weights, that are present for short durations, for instance when irrigation water is applied and enter the trays, or when drainage occurs post-irrigation). Hence, it was important to pre-process or de-noise the raw data in three stages: 1st: Removal of the extremely noisy or spiky points that occurred during irrigation, prior to converting the load cell observations into evapotranspiration profiles (ET_r_); 2nd: Filtering the remaining noisy data points from the variations in ET_r_ that corresponded with the sudden changes in ambient conditions; 3rd: Smoothing the resultant ET_r_ profiles.

For the first stage, a wavelet based approach, Maximum Overlap Discrete Wavelet Transform (MODWT) was implemented, since wavelets can inherently capture localized information in any signal through the scale and translation parameters in the wavelet function, that control the location of the wavelet at any given point of time. Hence, wavelets are regarded as one of the most suitable methods to de-noise a time series that contains high frequencies in short time periods [29, 30], i.e. those caused during irrigation events for instance. Here, MODWT was preferred to Discrete Wavelet Transform method, since MODWT allows direct correspondence between the time series and its decomposition at each level, unlike Discrete Wavelet Transform [31]. The Daubechies wavelet (db2) was executed on the load cell time series of each genotype up to a maximum of eight levels, and the apt level of decomposition was chosen such that data was de-noised without substantial loss of information i.e. only the high frequency coefficients are removed and the remaining variations are retained. In wavelet decomposition, the coefficients get smoother as the wavelet level increases [32]. Therefore, through trial and error, it was found that the smoothness of the data series at the third level seemed most appropriate (for eliminating anomalies from irrigation events) [33, 34]. The locations of the boxplot-based outliers in the MODWT coefficients of the first three levels were then retrieved and the corresponding observations were removed from the dataset. The missing values were linearly interpolated, and differenced at lag 1 to obtain the ET_r_ profile with 15 min frequency, of each genotype.

For the second stage, it was essential to first distinguish random noise from fluctuations caused by changes in environmental variables, since frequent changes in temperature (T in °C), relative humidity (RH in %), VPD (in kPa), wind speed (WS in ms^−1^) and solar radiation (RAD in MJm^−2^15 min^−1^) under non-controlled conditions, can lead to sudden changes in ET_r_ [19]. Therefore, although complete de-noising of the load cell data could be done through the wavelet method, the Penman–Monteith evapotranspiration, ET_0_ (mm/15 min) was chosen as the benchmark to selectively filter the ET_r_ profiles. ET_0_ was computed every 15 min, using the weather variables collected from the Campbell sensors installed at the LS platform. The Penman Monteith equation (ET_0_) was adapted (Eq. 1) to represent the 15 min interval by dividing the constant 900 by 96 (i.e. the frequency per day), as suggested by Zotarreli et al*.* [35].

1 $$E{T}_{o}=\frac{0.408\Delta \left({R}_{n}-G\right)+\gamma \left(\frac{900/96}{T+273}\right)WS \left({e}_{s}-{e}_{a}\right)}{\Delta +\gamma \left(1+0.34 WS\right)}$$

where

2 $$\Delta =\frac{4098\left[0.6108 exp\left(\frac{17.27\times T}{T+237.3}\right)\right]}{{\left(T+237.3\right)}^{2}}$$

3 $${e}_{s}=0.6108 exp\left[\frac{17.27\times T}{T+237.3}\right]$$

4 $${e}_{a}={e}_{s}\left[\frac{RH}{100}\right]$$

where, *ET*_*o*_ = reference evapotranspiration (mm 15 min^−1^), *R*_*n*_ = the net radiation flux (MJm^−2^15 min^−1^), *G* = the sensible heat flux into the soil (MJm^−2^15 min^−1^), $\gamma$ = psychrometric constant (kPa °C^−1^), $T$ = temperature (°C), *WS* = Wind Speed (ms^−1^), ${e}_{s}$ = saturation vapor pressure (kPa), ${e}_{a}$ = actual vapor pressure (kPa), $\left({e}_{s}-{e}_{a}\right)$ = saturation vapor pressure deficit (kPa), $RH$ = relative humidity (%).

The ratios of observed ET_r_ to ET_0_ were subsequently calculated, and the threshold ratio of 1.0 for day-time (solar radiation greater than 0) and 1.5 for night-time (solar radiation equal 0) were empirically identified for the filtering process. Every observation which resulted in a ratio beyond 1.0 or 1.5, for day and night-time respectively, was filtered out before smoothing the ET_r_ time-series. Filtered data were then interpolated as explained above. An average of 110 data points was removed per sector. So, given that each sector comprised 1440 data points in total (15 days × 96 values/day), 7.6% values were removed per sector.

In the third stage, the thresholded ET_r_ profiles were smoothed using the cubic smoothing splines [36]. In this study, the smoothing parameter, λ was determined by the generalized cross-validation method and 20 knots were used in fitting polynomials to the ET_r_ time series of all the sectors. The generalized cross-validation (GCV), which reportedly generates the optimal degree of smoothing [37] is implicitly included in the R routine used for implementing cubic smoothing splines [36–39]. Several previous researches [40, 41] have also shown the suitability of cubic smoothing splines for uniformly sampled time series analysis. The chosen set of procedures also enabled computational-convenience in curating raw data, an essential aspect of HTP [42].

#### Extraction of TR from ET_r_

Each smoothed ET_r_ profile was submitted to Eq. 5 to calculate Transpiration (T_r_) from ET_r_. The Leaf Area Index (LAI) was calculated from the empirical relationship that correlates the observed leaf area (LA) values with those obtained from the 3D laser scanners of the LS platform [13]. The observed LA was also calculated from those regressions, empirically such that Observed LA = 2.5* 3DLA, where 3DLA is the three dimensional leaf area, i.e. the measurement of the leaf area taken by the scanner. The value of β in Eq. 5 was taken as 0.463 which is considered valid for majority of agricultural canopies [43]. The T_r_ values thus obtained were converted to Transpiration Rate ($\mathrm{TR}$, i.e. the transpiration per unit of leaf area) by dividing T_r_ of each day with the corresponding Observed LA (Eq. 6). At the LS, LA is scanned every two hours, however, the maximum LA of each day was used, assuming that the changes in LA and its effect on TR in the course of a day would be close to negligible.

5 $${T}_{r}=\left[\left(1-{e}^{-\beta \times LAI}\right)\right]\times {ET}_{r}$$

6 $$TR=\frac{{T}_{r}}{Observed LA}$$

#### Preliminary analysis of TR vs VPD

After the TR profiles were extracted, the quality of those values (obtained through preprocessing) was examined to confirm that the differences between the three putatively known groups (wild, highTE and lowTE) were retained, and remained consistent across the days. Therefore, in light of the desired water-saving trait, a holistic analysis between the three groups was carried out using maximum TR and the corresponding maximum VPD values of each day, as per the conventional approach to identifying genotypic differences [12, 44]. First, maximum TR was regressed on maximum VPD, individually for each group, and the overall trend of the relation was studied. Subsequently, to test the significance of the differences among the groups and the consistency of those differences across the crop growth duration, a two-way analysis of variance (ANOVA) between the groups and the days was carried out using the maximum TR values. The ANOVA model used was: ${y}_{ijk}=\mu +{G}_{i}+{D}_{j}+{G\times D}_{ij}+{e}_{ijk}$, where ${y}_{ijk}$ is maximum TR values of the genotypes present in *i*^*th*^ group, *j*^*th*^ day and *k*^*th*^ replicate. ${G}_{i}$, ${D}_{j}$, ${G \times D}_{ij}$ and ${e}_{ijk}$ denote the fixed effects of the *i*^*th*^ group, the *j*^*th*^ day, the interaction effect between the *i*^*th*^ group and the *j*^*th*^ day and the residual error term respectively. To further explore the between-group differences, the same model was implemented between each pair of groups and the p-values were used to determine the presence of statistically-significant differences.

This analysis was however, limited to the three known groups (wild, highTE, lowTE) because it was expected that the highTE and lowTE groups could differ in their TR responses under high VPD conditions, and the wild relatives would differ from the cultivated chickpea. The remaining 12 genotypes i.e. the cultivated checks were not included, since there was no prior information about the expected response to high VPD. Therefore, once the quality of the extracted TR profiles was authenticated in these three groups, clustering and feature selection was performed to enable discretization of all the genotypes based on the inherent characteristics in their response to VPD, and independently from their putative a priori grouping.

### Clustering genotypes using TR features

#### TR time Series feature extraction

Feature extraction was performed as part of the second objective. A total of fifteen features (Table 1) per sector and per day were extracted considering the diurnal cycle in Patancheru, Hyderabad during the period of data collection: (i) sun rise occurred between 06:30 and 06:45 h and sun set between 19:15 and 19:30 h (here sunrise and sunset started when PAR sensor data started giving values either above or below 1 W m^−2^); (ii) the VPD effect was expected to mostly occur between 10:00 and 15:00 h.

**Table 1:** Feature description and abbreviation, computed for TR time series of each sector

Feature No	Description	Abbreviation
1	Total area under curve on each day	total.auc
2	Total area under curve between 07:00 and 19:00 h	auc.07.19
3	Proportion of area under curve during day time	auc.prop.07.19
4	Proportion of area under curve during night time	auc.prop.night
5	Slope of the curve from 19:00 to 23:45 h	slope.19.23.45
6	Maximum TR	maxTR
7	Slope of the curve between 6 data points before maxTR and maxTR (i.e. 90 min)	slope.maxTR.6
8	Slope of the curve between 00:00 and 07:00 h	slope.00.07
9	Slope of the curve from 07:00 h till it reaches maximum TR	slope.07maxTR
10	Curvature or angle of the curve at max TR	curvmaxTR
11	Area under curve between 10:00 and 15:00 h	auc.10.15
12	Proportion of area under curve between 10:00 and 15:00 h	auc.prop.10.15
13	Standard deviation of the TR values between 10:00 and 15:00 h	sd.10.15
14	Standard deviation of the TR values between 07:00 and 19:00 h	sd.07.19
15	Similarity between the ET_r_ profile of each sector and Penman Monteith ET	cos.sim.index

Features 1–5 described day and night TR profiles, features 6–14 represented the TR behavior during the highest VPD, radiation and temperature period of the day, and feature 15 described the similarity between the ET_r_ profile of each sector and ET_0_ computed using the cosine similarity index, cos.sim.index [45] (Table 1).

#### Clustering and feature selection

Genotype clustering and feature selection were also a part of objective 2, partitioned into three sub-objectives: (i) what is the optimal number of clusters inherently present among the genotypes; (ii) which genotypes cluster together (e.g. which cluster should be selected for crop improvement) and (iii) which features best distinguish the clusters (i.e. the proxy phenotypes).

(i) The mean of the 15 features (i.e. average of the four replications, and across all the days in the time series) of each genotype were used for clustering. The optimal number of clusters was first identified using the Dunn index, an internal cluster validation method. The index was computed based on the centroids obtained from the Euclidean distance matrix of the entire feature set [46] by varying the number of clusters from a minimum of two to a maximum of ten. The number of clusters equal to three, yielding the largest index value was chosen as optimal, and the genotypes were clustered using the unsupervised Random Forest (uRF) algorithm implemented via rfUtilities package in R [47].

(ii) The RF clustering method actually utilizes a user-defined conventional clustering procedure to cluster the RF dissimilarity matrix (comprising a dissimilarity measure between every two samples) and not the raw data [24, 48]. In this work, PAM (Partitioning Around Medoids) was used for clustering the RF dissimilarity matrix [24]. RF dissimilarity is also proven to be scale-invariant and preferred to Euclidean distance-based clustering, although RF dissimilarity and Euclidean distances are shown to correlate closely [49]. To examine the uRF model performance, Out-Of-Bag (OOB) error rate i.e. the proportion of incorrect predictions of the samples (genotypes) left out during bagging (model training) was used. And, to obtain the model with the minimum OOB error rate, uRF was trained through tenfold cross validation by sequentially varying the ‘*mtry*’ parameter (the number of random features selected during model training), from one through ten, while the ‘Number of trees’ was selected as 500 [24].

(iii) The features were ranked and then the most informative features were selected, based on the Mean Decrease in Gini coefficient (MDG) that quantifies the degree to which a feature contributes to node homogeneity in a tree [50]. Higher the MDG, higher is the loss in node homogeneity i.e. poorer is the decision-making ability in the absence of that feature [51]. Therefore, the variables with the highest estimates of MDG were selected as the most important features. On many occasions, such feature selection requires human intervention, and tends to be subjective. Hence, change point analysis [52] was employed on the sorted vector of the MDG to facilitate systematic feature selection. The number of desired change points was restricted to one, such that the subset of features that lied in the higher ranking partition would be the most important [53]. Since MDG is a local criterion and used during model training, once the model was trained (i.e. the clusters are identified) the importance of a feature in correctly predicting the OOB (or test) samples of a given class were estimated in terms of class-specific Mean Decrease in Accuracy (MDA). MDA tells the percentage reduction in overall prediction accuracy or increase in the OOB error rate, if a particular feature is permuted or excluded [24].

#### Estimation of heritability and statistical significance of the selected features

The biological significance and the genotypic variation of the selected features was then investigated using the broad sense heritability (H^2^) estimates, for crop improvement and breeding applications [23]. The H^2^ for each feature (each feature vector contained the average across all the days) was calculated as an estimate of its biological significance, using the formula: $\sigma$ ^2^(${\varvec{g}}$) / $(\sigma$ ^2^(${\varvec{g}}$) + $\sigma$ ^2^(${\varvec{e}}$)), where $\sigma$ ^2^(${\varvec{g}}$) and $\sigma$ ^2^(${\varvec{e}}$) is the genotypic and residual variances, respectively, that were derived from the linear model: ${\varvec{y}}=1\mu +{{\varvec{Z}}}_{{\varvec{g}}}{\varvec{g}}+{\varvec{e}}$, where $1$ is a vector whose all elements are one, and $\mu$ is the grand mean. In this model, the average of the feature values across all the days, ${\varvec{y}}$, were modeled in terms of the genotypic values, ${{\varvec{Z}}}_{{\varvec{g}}}{\varvec{g}}$, (where the vector ${\varvec{g}}$ contains the random genotypic effects and ${{\varvec{Z}}}_{{\varvec{g}}}$ is the associated design matrix) and the residuals, ${\varvec{e}}$. Thus, H^2^ represented the proportion of variance in the feature described by the genotypic effect.

Analysis of Variance (ANOVA) was then performed within each selected feature (the model used was similar to that of heritability estimation) and the p-values were examined for the presence of statistically-significant genotypic effect. The genotypic differences were subsequently examined within each selected feature by the pair-wise comparison of genotypes, based on the Tukey-HSD criterion [54].

### Analyzing sensitivity of TR to the environmental variables

This third objective aimed at identifying which among the different environmental variables had the maximum influence on the TR response. For this, a multi-output feed-forward Rectified Linear Unit (ReLU) network with 1 hidden layer containing 5 units was built using the *nnet* function in R [55] for three response variables (i.e. average TR profile of each of the three clusters) and five predictors (T, RH, VPD, RAD, WS). These predictors took 96 values on each day (one value every 15 min), and the values of the same time interval were averaged across 15 days. The number of epochs (i.e. the number of times the entire training data is presented to the model for learning) needed to prevent overfitting and the best-fit model were selected through cross-validation [56], in which 70% and 30% of the total dataset were used as the training and validation sets, respectively. In this analysis, the training data set was not partitioned into multiple batches, hence the number of iterations needed to complete an epoch was 1. The performance of the NN for each epoch was examined after the training process in terms of mean squared error (MSE) of the values predicted for the validation set. The Broyden–Fletcher–Goldfarb–Shanno (BGFS) optimization method was used to adapt the model weights during the training phase [55], and cross-validation was repeated till a maximum of 1500 epochs to select the model with the minimum validation loss (i.e. minimum MSE) as the best-fit model [57]. Subsequently, correlation coefficient (R) between the predicted TR and the input TR of the validation set was also evaluated from the best-fit model results, followed by further exploration of the causal relationships between the predictors and TR of each cluster.

The same NN model was used to identify the causations i.e. increase or decrease in the TR is caused by which weather variable(s), and whether that change is linear, non-linear or there is no change (= no effect of a particular predictor) through sensitivity analysis. Sensitivity analysis enables to explore every bivariate relationship by keeping the other variables constant at a certain rate, such that only the selected predictor is allowed to vary across its full range and the response variable is predicted. A general approach is to keep the variables constant at their means, or vary between mean + -1SD [58]. However, to enable exploration of a wider range of sensitivity of TR (of each of the clusters) to the environmental variables, the predictors were sequentially kept constant at different rates i.e. at 0, 20, 40, 60, 80 and 100% splits of their quantiles [59], that represented corresponding levels of environmental interaction. Once the predicted transpiration rates were obtained, for each predictor and at each split, the slope or the rate of change in TR for every bivariate pair was calculated in terms of percentage, to obtain the estimates of Sensitivity Index (SI) [58, 59]. Since the response to VPD was non-linear and the objective was to identify the VPD breakpoint in the TR response, the first linear slope was considered for SI estimation [60]. Finally, the VPD level at which the slope of the tangent line of each TR response curve approached zero, was recorded for each level of environmental interaction as the breakpoint at which maximum TR was attained. Thus, sensitivity analysis provided a quantitative tool for discretizing the genotypic differences in the water-saving trait, under varying levels of influence by other variables simultaneously.

## Results

### Preliminary analysis of genotypic differences from ET_r_ and TR profiles

**Fig. 2:**
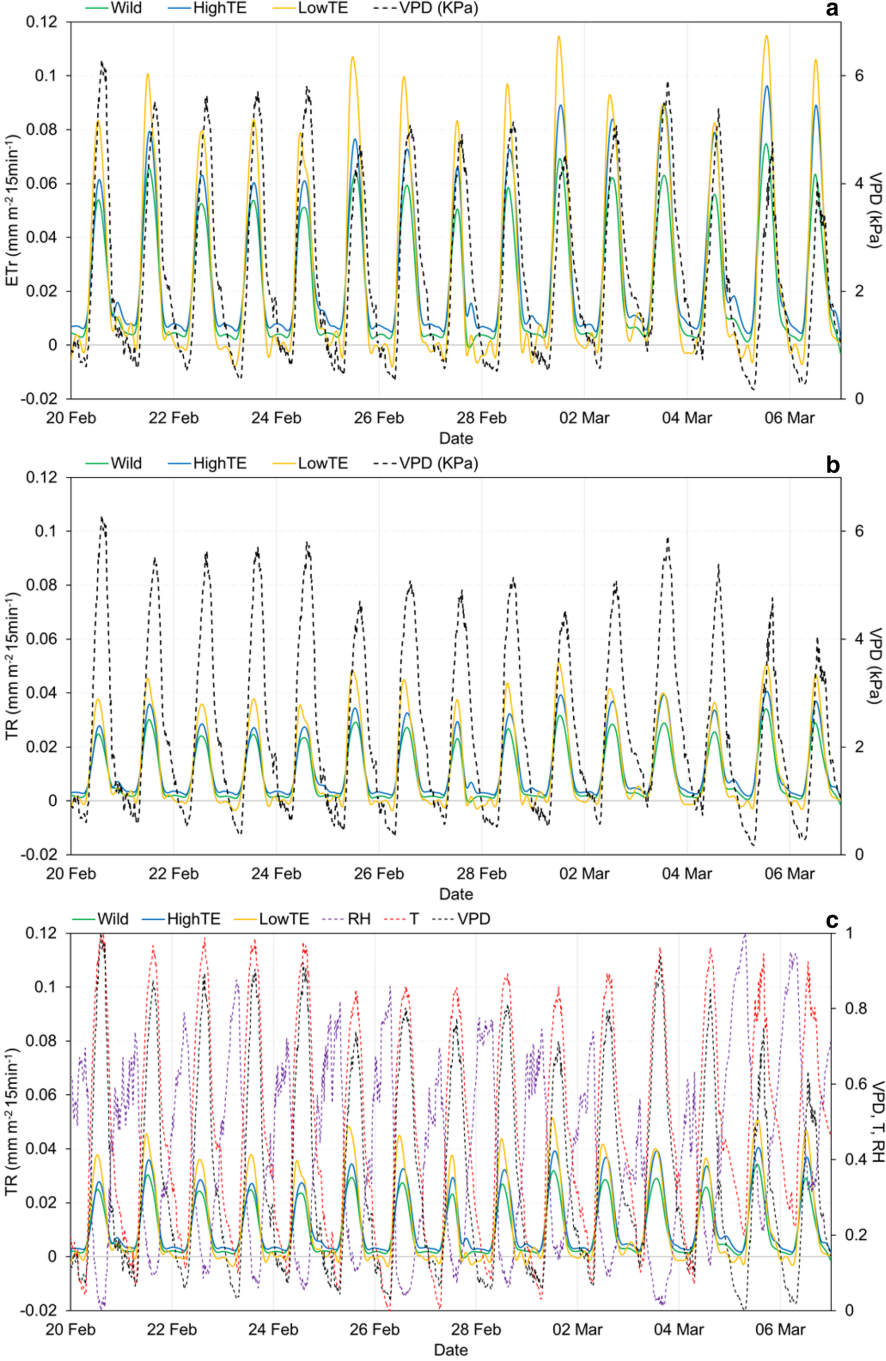
**a** Average ETr and **b** TR time series of wild, highTE and lowTE groups plotted with respect to VPD (kPa). **c** The normalized values of VPD, T and RH plotted along with average TR time series of wild, highTE and lowTE groups to enable simultaneous inspection of the time series variations

During the window of measurements considered in this work, plants were irrigated on 4 days (21st Feb, 25th Feb, 1st March and 5th March). Anomalous sector weight changes were detected and removed through MODWT (Fig. 1a). Hence, on converting the sector weights to ET_r_ profiles, although differences among genotypes were visible, data remained noisy around those days (Fig. 1b). After filtering ET_r_ values with respect to ET_0_ (Fig. 1c), and smoothing the ET_r_ profiles, the genotypic differences became distinct. For instance, the wild germplasm had a flatter response i.e. had lower water loss during high VPD conditions than the highTE and lowTE ones, which was expected [61]. In addition, this flatter profile was congruent with the hypothetical model of the water-saving trait, illustrated in Fig. 1d (adapted from Sinclair et al*.* [4]). A visual analysis of the average ET_r_ (Fig. 2a) and TR (Fig. 2b) time series of the three groups (wild, highTE and lowTE) with respect to VPD reinforced the above inference. The group of wild accessions had the lowest ET_r_ and TR, while the ET_r_ and TR of the lowTE lines were higher than that of both the wild and the highTE groups. This suggested a transpiration limitation under high VPD in the highTE group, also expected from the theory [1, 4] and also in concordance with Fig. 1d. Further, the intermittent fluctuations in the environmental variables (T, RH, VPD) and the TR profiles (Fig. 2c), and their possible correspondence e.g. a tendency for higher ET_r_ on days with the lowest VPD, not only showed the credibility of the proposed data processing procedure but also the sensitivity/ability of the platform, in capturing those subtle variations.

As a general pattern, the change in TR followed VPD within a day, while maximum TR was inversely proportional to maximum VPD, across the days. The negative slopes of a linear regression between maximum VPD and maximum TR of the wild (Additional file 1: Figure S1a), highTE (Additional file 1: Figure S1b) and lowTE (Additional file 1: Figure S1c) genotypes confirmed these inverse relation (p < 0.05; Table 2), besides revealing the increasing order of maximum TR’s sensitivity to VPD, from the wild (slope = −0.0042) to highTE (slope = −0.0053) to lowTE (slope = −0.0058) groups. As expected, the wild relatives differed significantly from the cultivated lines i.e. both highTE and lowTE. However, although both highTE and lowTE groups differed overall in their maximum TR, the difference between the two was not significant in every day of the testing period (since the interaction effect was not significant, p > 0.05). Hence, it could be inferred that either maximum TR was not sufficient in completely capturing the differences among the cultivated varieties or prior grouping information was not optimal with regard to the targeted trait.

**Table 2:** Results from two-way ANOVA conducted for all the three groups (Wild-highTE-lowTE) and between each pair of groups i.e. highTE-lowTE, wild-highTE and wild-lowTE to understand the significance of the effects of Day, Group and the interaction between Day and Group on the transpiration rate (TR) values

Sources of Variation	Wild-HighTE-LowTE					HighTE-LowTE				
	Df	SS	MS	F value	Pr(> F)	Df	SS	MS	F value	Pr(> F)
Day	14	0.03875	0.002768	68.49	< 2E−16***	14	0.02591	0.001851	14	< 2E−16***
Group	2	0.02152	0.010761	266.27	< 2E−16***	1	0.00021	0.000214	1	0.0242*
Day × group	28	0.0043	0.000154	3.8	1.11E−10***	14	0.00019	1.34E-05	14	0.9918
Residuals	1755	0.07093	0.00004			975	0.04092	0.000042	975	
Sources of variation	Wild-HighTE					Wild-LowTE				
	Df	SS	MS	F value	Pr(> F)	Df	SS	MS	F value	Pr(> F)
Day	14	0.02536	0.001811	42.965	< 2E−16***	14	0.02881	0.002058	54.788	< 2E−16***
Group	1	0.01644	0.016438	389.942	< 2E−16***	1	0.01331	0.013307	354.301	< 2E−16***
Day × group	14	0.00325	0.000232	5.503	2.26E−10***	14	0.00258	0.000185	4.913	5.88E−09***
Residuals	1245	0.05248	0.000042			1290	0.04845	0.000038		

*p-val < 0.05

**p-val < 0.01

***p-val < 0.001

### Identification of genotypic clusters and the most important TR features

**Fig. 3:**
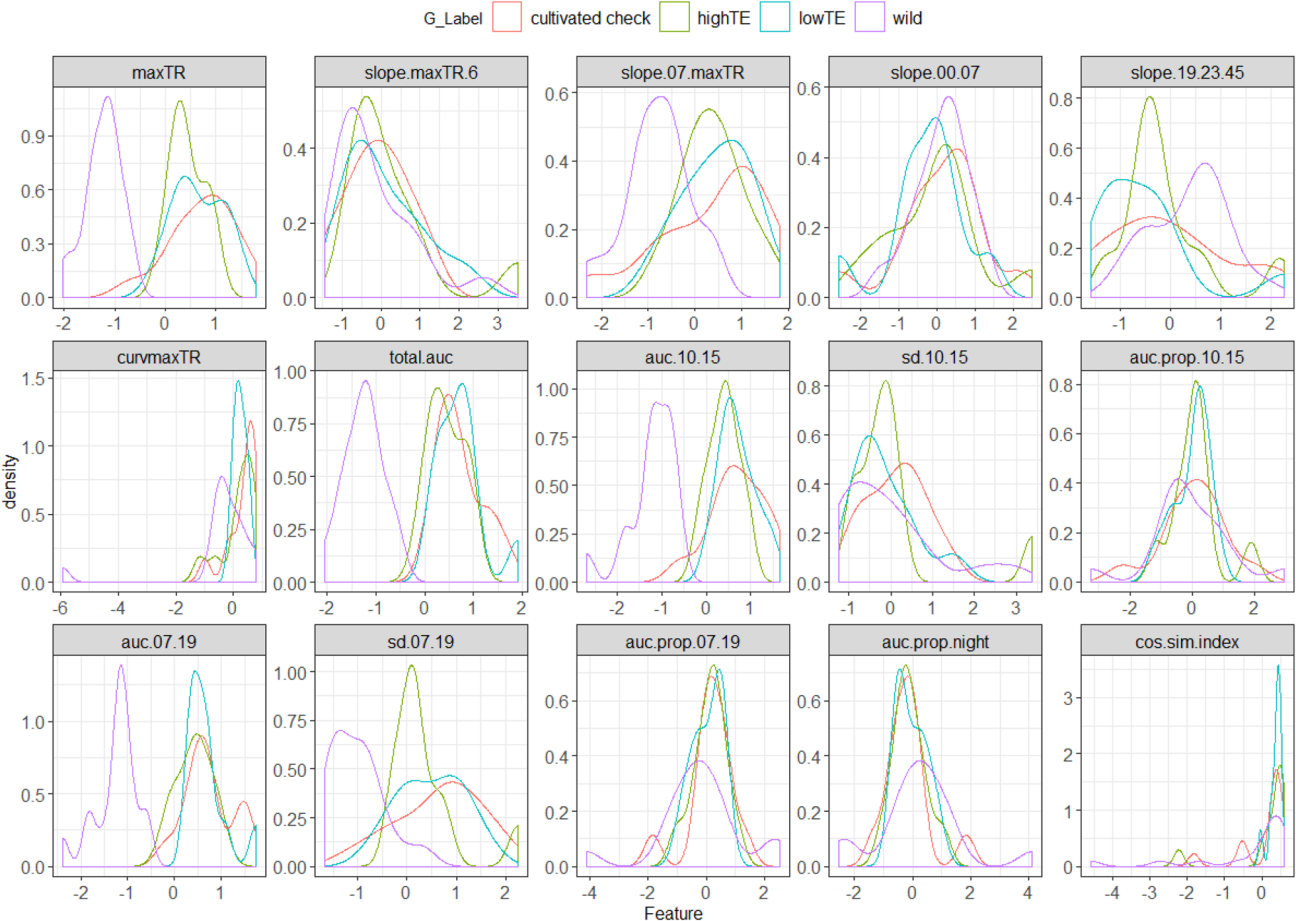
Density plots of the fifteen features extracted from the TR time series, that illustrate the differences in the distributions of the wild, highTE, lowTE and cultivated check varieties

The fifteen features extracted from the TR time series are illustrated through density plots in Fig. 3, which helped with four important inferences: (1) The probability density functions (PDFs) overlapped for many features implying similar feature values across the groups. Hence, based on an overall visual assessment of the individual distributions (PDFs), there appeared to be optimally two or three clusters; (2) The distribution of only the wild group was conspicuous across the feature set, besides having the lowest values thereby implying better efficiency in limiting transpiration during high evaporative conditions; (3) The overlapping PDFs, visible in almost every feature (e.g. maxTR, total.auc, auc.07.19 and auc.10.15—‘auc’ is the area under the curve), largely belonged to the highTE, lowTE and the cultivated checks. This implied that the composition of the clusters inherently present in the feature set might differ from the 4 groups known a priori; (4) The features that contained overlapping PDFs primarily belonged to the night time and were therefore less suitable for distinguishing the genotypes. Contrarily, the day time features like maxTR, auc.07.19, slope.07.maxTR, etc. showed maximum contrast among the PDFs and were expected to significantly contribute in optimally clustering the genotypes.

**Fig. 4:**
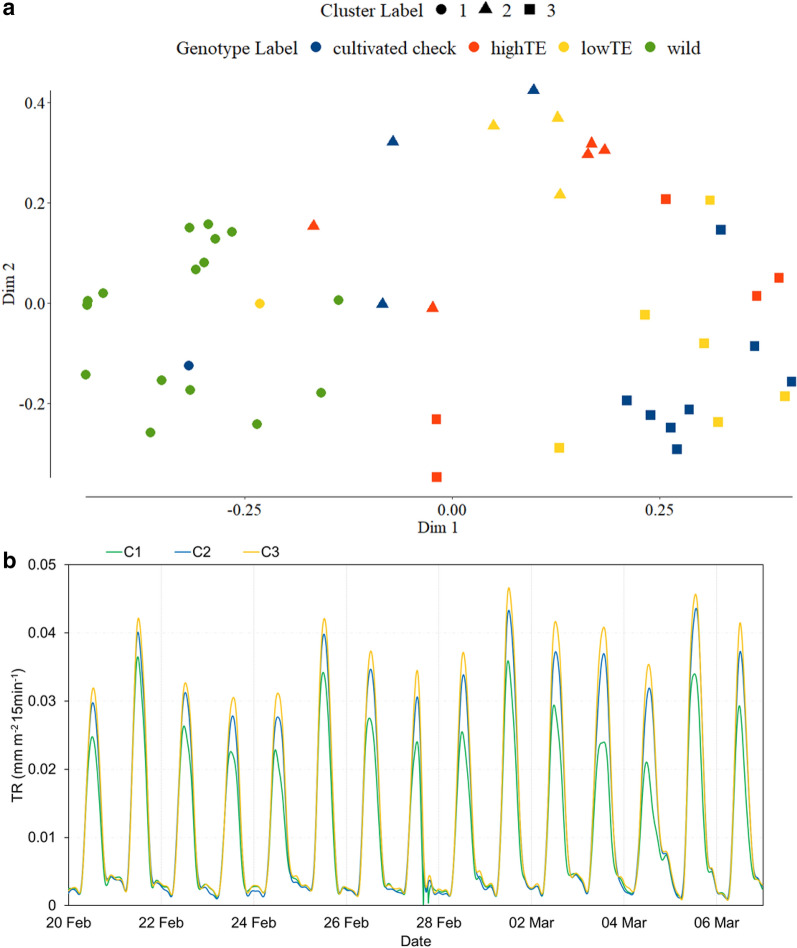
**a** Genotypic clusters obtained through random forest clustering are plotted on the first two dimensions of the Multi-Dimensional Scaling (MDS) plot, that also denotes the a priori label information of the genotypes. **b** The average TR time series of the three clusters (C1, C2 and C3) are shown from 20.02–06.03.2017

The Dunn index values (Additional file 1: Figure S2) confirmed that the optimal number of clusters was three, which validated the first intuitive inference made from Fig. 3. Subsequently, the three clusters (Fig. 4) obtained from the uRF (unsupervised Random Forest) model with the least OOB error rate of 6.2% (Additional file 1: Figure S3) i.e. with a prediction accuracy on new test data of 93.77%, were inspected to identify the genotypes that clustered together (Fig. 4a). The average TR of each cluster was also plotted (Fig. 4b) to visualize the differences in their TR profiles. Interestingly, all the wild chickpeas belonged to the first cluster (C1) and C1 had the least TR across the entire duration of the experiment, thereby substantiating the second inference from Fig. 3. With regard to the cultivated lines, a majority of the lowTE varieties (i.e. 6 out of 10) were found in the third cluster (C3) together with most of the cultivated checks (i.e. 8 out of 12), while highTE lines seemed dispersed between C2 and C3. Additionally, the TR values of C2 were consistently lower than C3, the cluster with the highest TR. These results corroborated the third inference from Fig. 3.

**Fig. 5:**
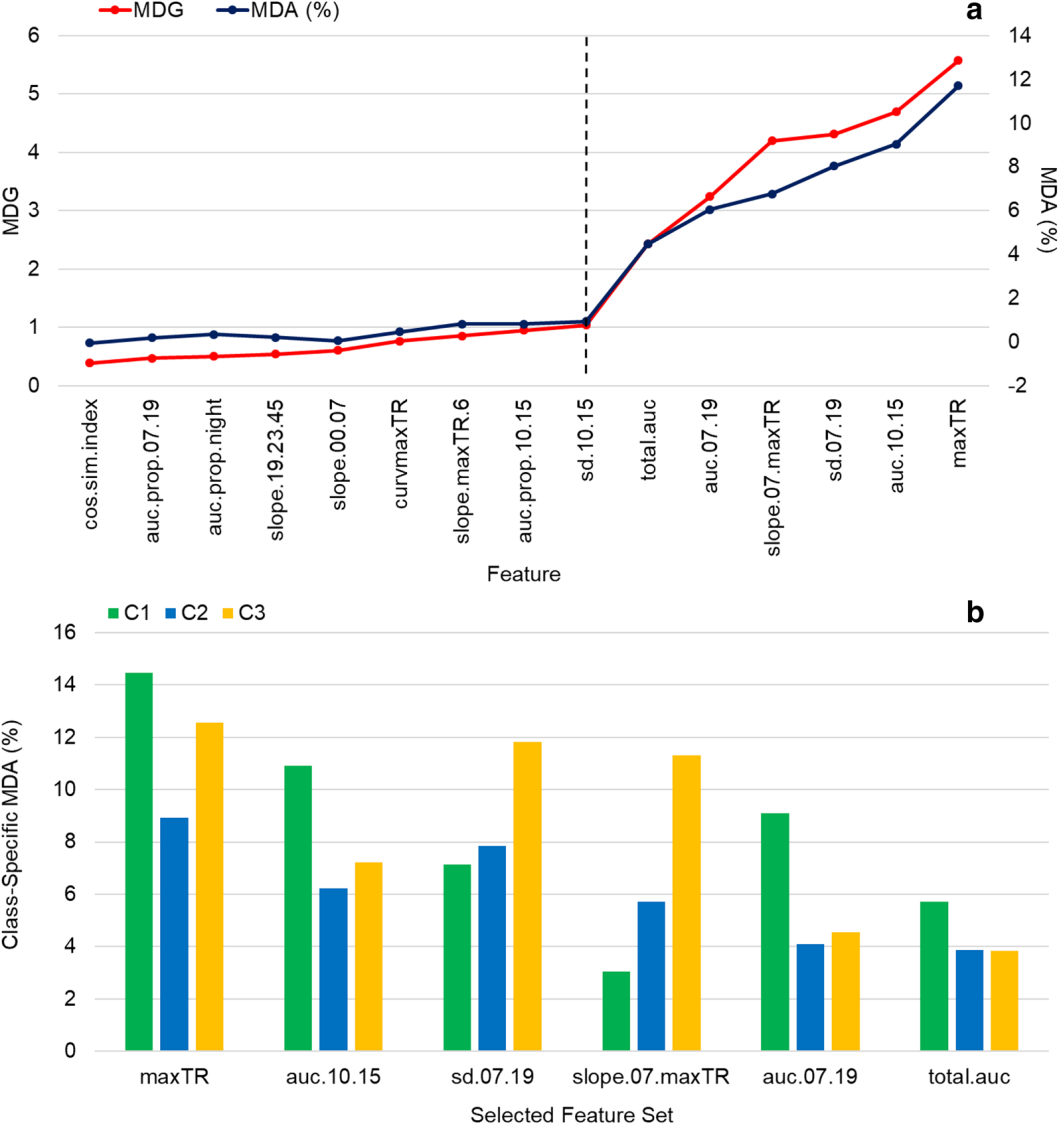
**a** The Mean Decrease in Gini (MDG) and the Mean Decrease in Accuracy (MDA, %) estimates obtained for each feature from the best-fit random forest model are plotted in the increasing order of MDG, and the change point (black dotted line) demarcates the set of least important features from the most important ones. **b** For the most important features i.e. those with the highest MDG and MDA in Fig. 5a (selected feature set), the corresponding class-specific (i.e. for each cluster, C1, C2 and C3) MDA are shown

Finally, the mean decrease in Gini (MDG) values of the day time features (maxTR, auc.10.15, sd.07.19, auc.07.19, slope.07.maxTR, total.auc) were much higher than others, implying higher importance in making those clusters (Fig. 5a), and validating the fourth inference from Fig. 3. The corresponding overall mean decrease in accuracy (MDA) values (Fig. 5a) of the selected feature set further ascertained that excluding those features had the highest impact on the overall classification accuracy of the uRF model. From the class-specific MDA of the selected feature set (Fig. 5b), exclusion of maxTR, auc.10.15, auc.07.19 and total.auc reduced the prediction accuracy of the C1 genotypes by ~ 14.5, 11, 9 and 6%, respectively. This meant that these features of the transpiration under high VPD conditions very strongly characterized the genotypes of C1. Similarly, accurate prediction of the C2 and C3 genotypes could mostly be ascribed to maxTR, sd.07.19 and slope.07.maxTR. Thus, the six selected features could be aptly regarded as the proxy phenotypes that best discretized the genotypes with respect to the differences in their transpiration response to increasing VPD conditions, the desired trait.

### Analysis of genotypic diversity within each selected feature

**Fig. 6:**
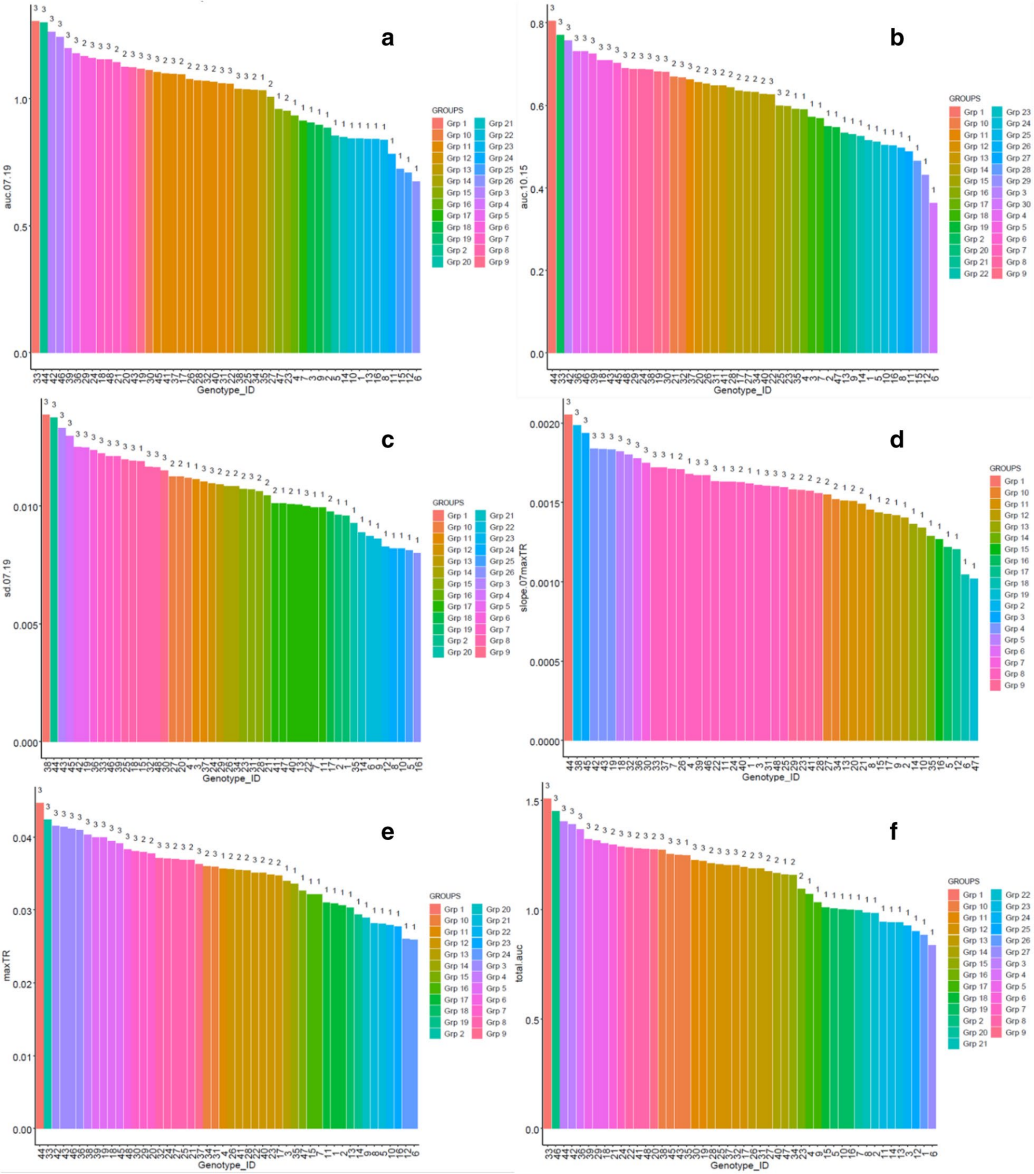
The number of distinct p-value (> 0.05) groups obtained from Tukey-HSD pairwise comparison of the genotypes for each selected feature is illustrated. The feature values are sorted from highest to lowest, and hence the X-axis labels (denoting the Genotype_ID) are sequenced accordingly. Each spectrum of feature values is also annotated with the corresponding cluster label of the genotypes

As shown in Fig. 6, the number of distinct p-value groups obtained from the Tukey-HSD pair-wise comparison of genotypes within a feature, was the highest i.e. 30, for auc.10.15. It was followed by total.auc, auc.07.19, sd.07.19, maxTR and slope.07.maxTR with 27, 26, 26, 24 and 19 statistically distinct groups, respectively. Thus, these results showed a very large range of diversity in the traits related to the TR response to increasing VPD, temperature and radiation in this set of genotypes, and the maximum diversity could be captured during the peak conditions. It was further noted that genotypes belonging to C1 and C3 were predominantly found in the lower and higher end of the spectrum of each feature, respectively, while the central portion comprised varieties from both C2 and C3. These results, therefore augmented the previous inference i.e. genotypes in the first cluster possessed higher water-saving characteristics than others during high evaporative demands. In terms of heritability estimates, five out of the selected features had H^2^ greater than 0.5 viz. total.auc (0.81), auc.07.19 (0.76), slope.07.maxTR (0.61), auc.10.15 (0.58) and maxTR (0.56).

### Analysis of the sensitivity of TR to the environmental variables

**Fig. 7:**
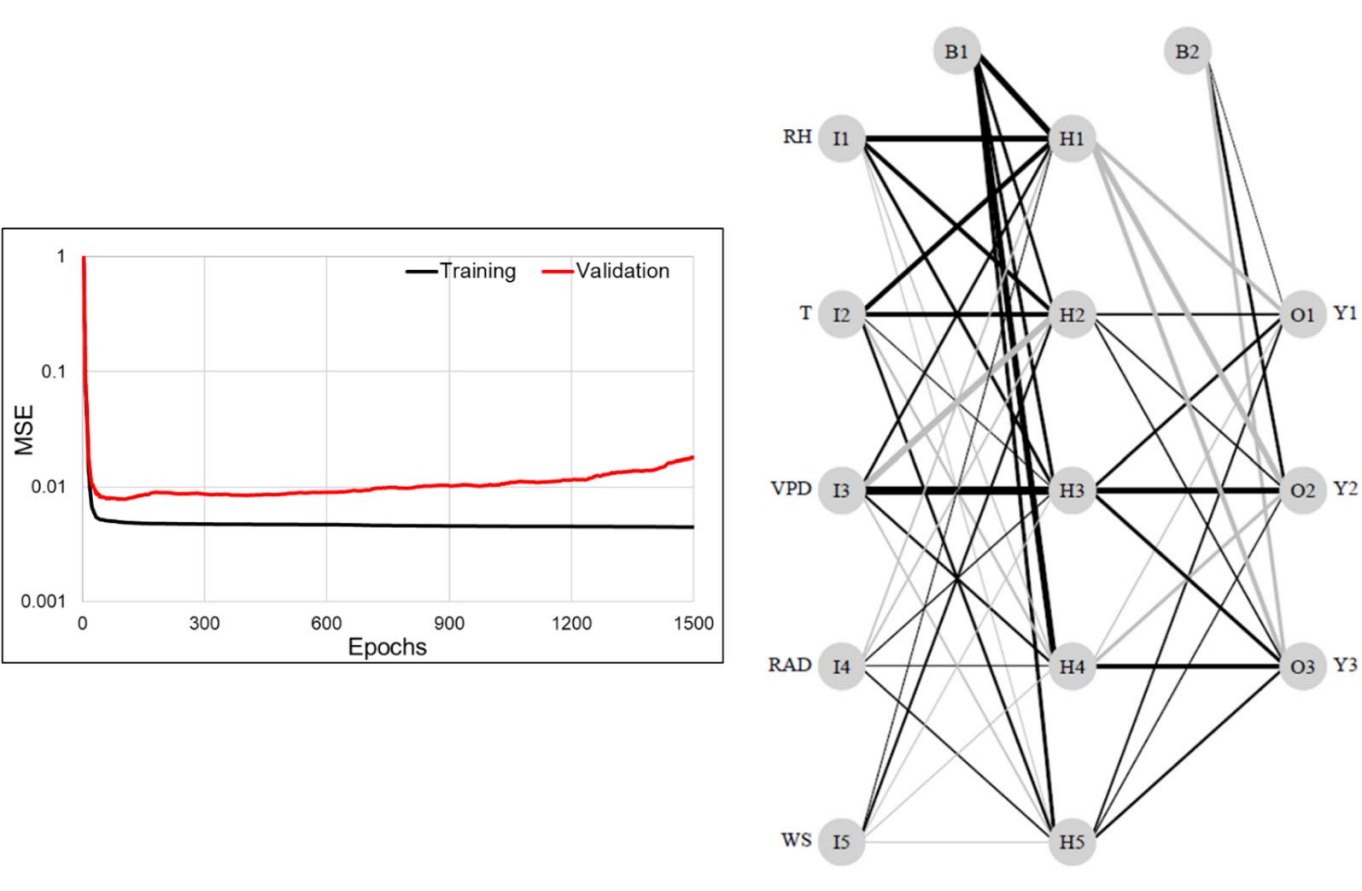
**a** The Mean Squared Error loss observed during Neural Network cross-validation, for both the train and test set at each epoch, where maximum epoch size is 1500 and **b** the Neural Network architecture of the best-fit model between the environmental variables (Relative Humidity-RH, Temperature-T, Vapour Pressure Deficit –VPD, Solar Radiation-RAD and Wind Speed-WS) and the average TR time series of cluster C1 (Y1), C2 (Y2) and C3 (Y3). The thicker lines imply variables with greater impact on the TR values than the thinner ones, and black lines indicate positive influence while grey imply negative effect. The neurons in the input, hidden and output layers are annotated with I, H and O, respectively. The bias neurons are B1 and B2, and can be considered similar to intercepts in linear regression models

**Fig. 8:**
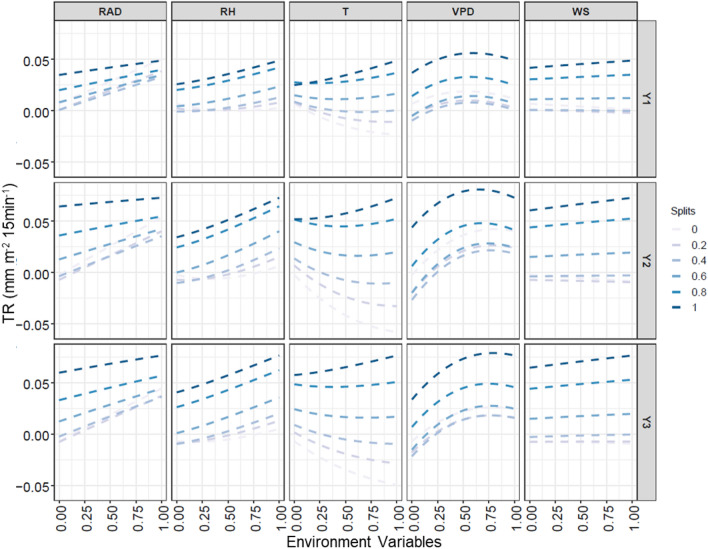
Results of the sensitivity analysis illustrating the predicted average TR (mm m^−2^ 15 min^−1^) profiles of C1 (Y1), C2 (Y2) and C3 (Y3) obtained with respect to changes in each of the environmental variables, when the simultaneous influence of the remaining variables progressed from minimum (split 0) to maximum (split 1). The values shown on X-axis are normalized between 0–1, denoting transition from minimum to maximum

**Fig. 9:**
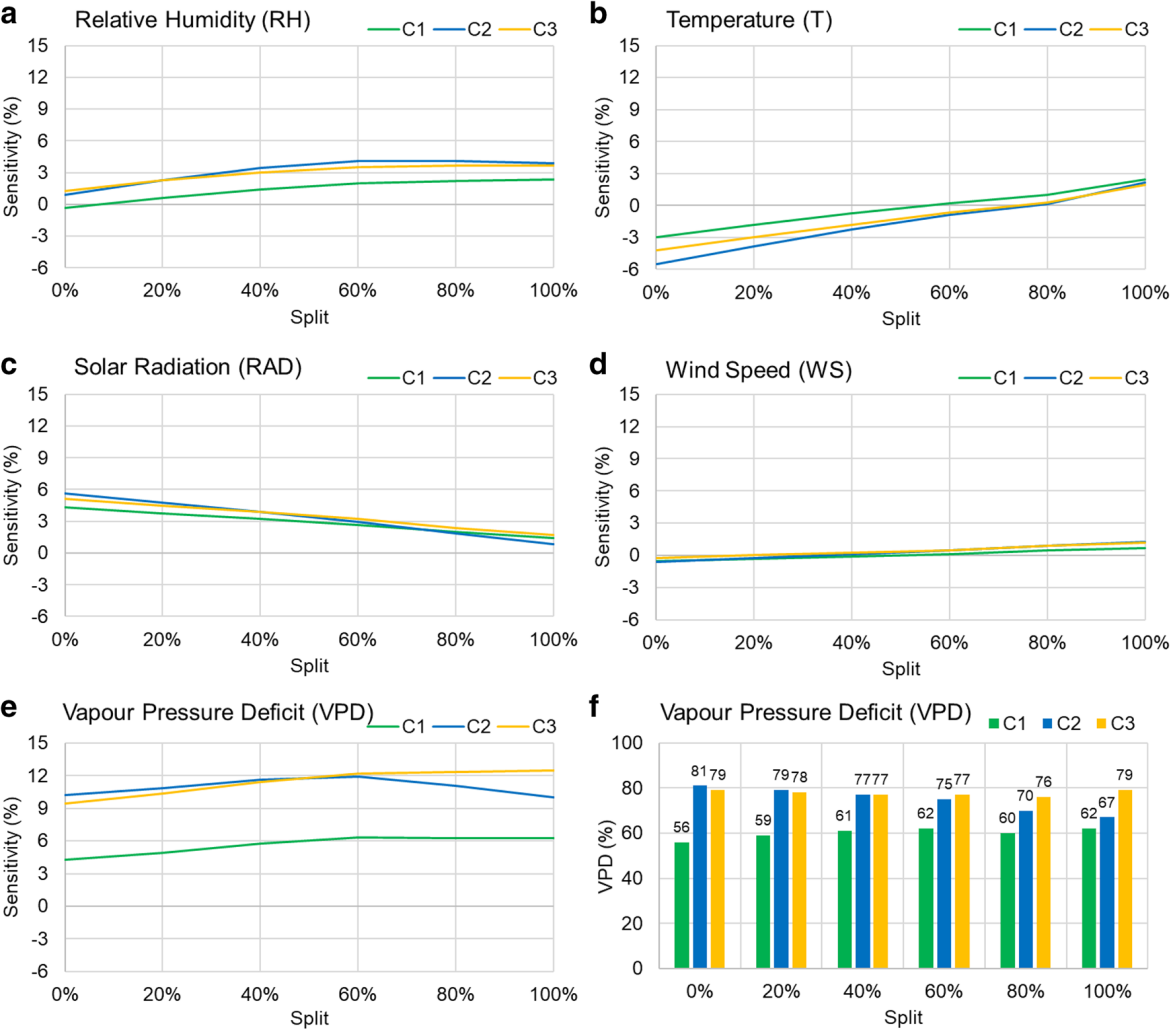
**a**–**e** The slopes (in %) of the TR profiles obtained through sensitivity analysis represent the Sensitivity (%) of TR (of C1, C2 and C3) to each environmental variable across the entire split horizon i.e. 0–100%. **f** The levels of VPD (%) representing the breakpoints at which maximum TR is restricted are plotted for each of the clusters (C1, C2 and C3) and at each level of environmental influence i.e. split 0–100%

The NN cross-validation results and the best-fit NN used to model the relationship between the environmental variables and TR of C1, C2 and C3 are illustrated in Fig. 7. From the variation in mean squared error (MSE) corresponding to each epoch and particularly the upward trend of the validation set loss curve (Fig. 7a), the model overfitted the training set beyond the 91st epoch. Hence, the best-fit model was chosen at epoch 91, which had the minimum validation MSE (0.0078) and the corresponding training MSE was 0.0049. The R between predicted TR and the input TR of C1 (Y1), C2 (Y2) and C3 (Y3) of the validation set were 0.931, 0.944 and 0.953, respectively. Visual inspection of the best-fit NN model (Fig. 7b) revealed that VPD, RH and T had the strongest positive effects (in descending order) on TR followed by RAD and WS with grey/black lines representing negative/positive weights and line thickness representing the strength of the connection or effect of the particular predictor on the response(s). It was also noticed that C2 and C3 were more influenced by the changes in ambient conditions than C1. The sensitivity analysis plots (Figs. 8, 9) supported these inferences, both quantitatively and precisely at different levels of environmental interaction effects.

From the bivariate relationships (Fig. 8), the non-linear response of TR to VPD differed from the other predictors, most of which had linear, positive relationships with TR. These differences in the sensitivity of TR to the weather variables were quantified for each cluster, using the slopes of the individual responses, and shown in Fig. 9a–e. The sensitivity of C1 and C3 were consistently the lowest and highest (particularly during the high VPD conditions i.e. beyond the stage where VPD reached 60% of its maximum values), respectively, as already found from the previous results e.g. Figure 4b. Among the linearly influencing variables (RAD, RH, T and WS), only the effect of T was found unique, since TR response to T transitioned from negative to insignificant to positive, as the simultaneous influence of others (i.e. RAD, RH, VPD and WS) progressed from minimum (split 0) to maximum (split 1). This meant that increase in T led to increased water loss only when other variables had greater influence (i.e. more than 80%). These four variables however did not significantly contribute to identifying cluster-specific differences in the TR characteristics, due to almost overlapping SI profiles of C1, C2 and C3 (Fig. 9a–d). The only variable that distinctly identified the TR characteristics among the clusters, was VPD (Fig. 9e). On inspecting the non-linear VPD-TR relationship it was further observed that clusters had reached respective maximum TR levels before VPD reached its maximum. The increase in TR was found linear only until a certain level of VPD, beyond which further rise in VPD led to decline in TR, thereby breaking the linearity (also clearly noticeable in Fig. 8). The breakpoints for all the clusters and at each level of environmental influence are presented in Fig. 9f. The three clusters showed three distinct patterns in the shift of their breakpoints, as the influence of other environmental variables advanced from minimum to maximum. While the breakpoint for C1 gradually increased from 56 to 62%, that of C3 was found almost consistent around 79%. These values shown are considering the maximum VPD experienced as 6.29 kPa, the 100% VPD level. The detail of VPD breakpoints for each cluster at each level of influence of other environmental variables (% split) are shown in Additional file 1: Table S3. This probably showed the resistance or independence of C3 to the influence of other variables, besides its evidently sharp sensitivity to VPD. In case of C2, the breakpoint surprisingly reduced from 81 to 68%, which proved that the TR response characteristics of C2 were closer to C1, under peak VPD conditions. Thus, this NN-based modeling of causal relationships helped quantify the genotypic differences in terms of the VPD levels where TR restriction occurred (Fig. 1d).

## Discussion

In this work, load cell data of a diverse chickpea population (wild, highTE, lowTE and cultivated check varieties) were used to develop a generic data analysis framework to characterize the transpiration response profiles to increasing VPD (Fig. 1d), using HTP data from a chickpea experiment carried out outdoors and under high VPD conditions. Major results obtained from this work were: (i) The data analysis framework generated highly relevant and reliable TR profiles, in line with an ET_0_ benchmark; (ii) The feature selection algorithm of the framework revealed additional key features of the TR response to VPD than the usual maximal TR values, and these could be used in crop selection; (iii) The framework also clustered the genotypes in a highly relevant way and allowed a detailed and quantitative genetic analysis of the identified features, showing high genotypic variation and high heritability of the main TR response features; (iv) Among the various environmental variables eliciting the TR response, VPD had by far the highest influence. These topics are further discussed below.

### Relevance of the framework-generated TR profiles

The data analysis framework generated highly relevant transpiration profiles, and in this smoothing process, the use of ET_0_ as a benchmark for the ET_r_ profiles was indeed crucial and showed that subtle variations could be retained. The choice of genotypes was such that genotypic differences were expected in the diurnal transpiration profiles, after converting the load cell values to ET_r_ profiles (as shown in Fig. 1b, c). Indeed, subsequent analyses delineated genotypic differences in transpiration response patterns, where the wild relatives transpired the least, whereas the lowTE ones transpired the most, as expected [1]. The influence of maximum VPD values on maximum TR across different days (Fig. 2c; Additional file 1: Figure S1) also remained clearly visible after preprocessing and conversion procedure. The negative relationship between maximum TR and maximum VPD suggested that transpiration declined in all genotypes when VPD reached high level. This deduction was congruent with previous studies that have also used linear regression and ANOVA to examine the relation between maximum TR and maximum VPD [12, 62]. A large difference between ET_0_ and ET_r_ was however observed. This could be explained by the fact that the meteorological variables used in the calculation of ET_0_ were collected from the cemented LS platform, and therefore not under standard meteorological station conditions. It could also have occurred because, as far as we know, the Penman–Monteith equation does not consider any VPD response, which influenced the collected transpiration profiles. Additional work would be needed to analyze the collinearity of TR profiles with ET_0_ across seasons with varying VPD levels. However, from such conventional ways of analysis, it was difficult to infer: (i) if it was sufficient to use only maximum TR for characterizing the latent genetic variation in the desired trait, (ii) the simultaneous effect(s) of other environmental variables in driving those response characteristics among genotypes, and (iii) the precise VPD levels that resulted in the restriction of maximum TR.

### Significance of feature engineering in identifying genotypic differences in the water-saving traits

The smooth TR profiles generated from the framework opened an opportunity to go beyond classifying genotypes merely on the basis of their maximum TR, by identifying additional features of the TR profiles. Therefore, feature engineering was adopted in this work to identify additional important features (besides maximum TR) that could characterize the genotypic differences in TR response. Machine learning-based studies that deal with large and complex datasets (e.g. raw load cells data/the TR time series) for specific pattern recognition (e.g. maximum TR restriction under high VPD), predominantly use features instead of the raw data [63]. This is because diligently engineered features most accurately represent the underlying structure of the data and can therefore yield the best interpretable model results [64]. However, in the context of HTP, feature-based analysis has rarely been considered for exploring continuous plant processes like the diurnal variations in TR. One of the best ways to generate prudent features is by the utilization of domain knowledge, such that the features explicitly characterize the desired patterns [65]. Among the fifteen features that were designed, six had the highest importance, especially auc10.15 that quantified the water saved during peak VPD hours. Those features also had high H^2^ estimates. Expectedly, the night-time features were less important as per the uRF model, with the least MDA and MDG estimates (Additional file 1: Table S2). These results were biologically highly relevant. Thus, through prudent feature engineering, the uRF model did indeed select variables that were intuitively the most important. The model also resulted in credible clusters, since C1 contained all the wild chickpeas and had the lowest TR, whereas C3 mostly comprised lowTE and cultivated check varieties with the highest TR. Accordingly, the selected feature set (Fig. 4a) maximized classification model accuracy (Fig. 4b), with an OOB error rate of 6% that showed a very good classification accuracy of 94%.

### Quantifying genotypic differences in the water-saving trait

Large genotypic differences were found for the six selected features (Fig. 6). These features also had high heritability, making them suitable in the context of a breeding program. From the SI estimates (Fig. 9) C1 was most resilient to all the variables, followed by C2 and C3. The differences in the sensitivities were however significant only in case of VPD, and from the breakpoints (Fig. 9f) it was noticed that the genotypes in C1 started limiting transpirational water loss at much lower percentage of maximum VPD than C2 and C3. Since C1 was mostly composed of the wild genotypes (i.e. 16 out of 18 C1 genotypes were the wild relatives), this result was consistent with previous analysis, conducted from transpiration data of the wild and cultivated germplasm [61]. It also reinforced the inferences obtained from feature-engineering based identification of genotypic differences (Fig. 4b) and showed that the wild chickpea germplasm indeed had the potential for water saving traits. On comparing the transition in the breakpoints of C1, C2 and C3, it was found that as the influence of other factors increased (i.e. during the high evaporative demand conditions), the breakpoint profile of C2 started converging towards C1. This finding precisely demonstrated that C2 (with more highTE and less lowTE genotypes) displayed a water-saving strategy closer to C1, which is consistent with the idea of a close relationship between transpiration restriction under high VPD conditions and higher TE [1]. These results further confirmed that besides having the least TR, the wild chickpea genotypes/accessions (contained in C1) elicit better and faster restriction of maximum TR to increasing VPD [66]. The exact values of VPD (kPa) corresponding to each breakpoint are given in Additional file 1: Table S3, considering the maximum or 100% VPD level as 6.29 kPa as observed during the experiment (refer Additional file 1: Table S1).

### Importance of VPD in the transpiration response patterns

Once the genotypes were optimally clustered (through uRF modeling), those differences were explored through sensitivity analysis on a multi-output NN, and the specific VPD levels at which the clusters restricted their maximum TR were discretized. In this exercise, the simultaneous effects of the other environmental variables were considered at varying levels (splits), unlike the typical regression analysis of transpiration versus VPD [44]. Sensitivity analysis revealed that an increase in VPD had a very strong positive effect on plant water loss irrespective of the influence of other variables almost until the 0.6 quantile, beyond which the response curve saturated, and further water loss was restricted. This non-linear trend aptly denoted the water-saving mechanism inherent in the genotypes. As per the sensitivity to T, plants restricted transpirational water loss even if temperature increased only when other factors had no or little effect. However, it is only when other factors reached values beyond 60% of their maximum values that TR restriction ceased to be effective, resulting in an increasing water loss; hence, the positive linear trend in predicted TR. These results were also consistent with other studies [11] showing the influence of temperature on the VPD response, and the fact that beyond a certain temperature threshold, transpiration loses its sensitivity to VPD and water losses increase. RAD and RH had similar linear effects, while WS did not seem to have any major influence on TR. The effects of T, RH, RAD and WS on TR were however secondary, compared to that of VPD. Although previous studies [15, 60] have reported similar findings, those were not quantified as shown in Fig. 9.

## Conclusions

Extracting information from raw HTP data is typically hindered by the unavailability of standardized and user-friendly procedures to convert raw data into usable and interpretable knowledge. Such complications are enhanced for complex functional traits like those discussed in this paper and that are difficult to phenotype under natural conditions. Therefore, this paper makes a novel attempt to facilitate systematic extraction of features of the water-saving trait through an ML-based quantitative diagnostic framework. It systematically dissects the subtle genotype-specific characteristics of transpiration regulation in response to VPD. Through this framework, six biologically relevant features were selected from the TR profiles, and three genotypic clusters were identified. C1 represented a cluster of water saving genotypes and comprised all the wild accessions, whereas C3 represented a cluster of water profligate genotypes containing a majority of low TE lines and cultivated checks. Such a genotypic differentiation was highly congruent with respect to the expected transpiration profiles of these genotypes. Additionally, most of the selected features had heritability greater than 0.5 and had great value for breeding. Auc.10.15 was one of the most important features for correctly classifying the genotypes, which also revealed maximum genotypic diversity. Thus, feature-based genotypic differentiation was highly relevant. Furthermore, weather predictors in the NN model could explain around 95% of the variability in TR response, with VPD having the highest importance. From sensitivity analysis on that model, plants were found to restrict transpiration before reaching the highest VPD level, although at different levels among genotypic groups. Precise identification of those levels showed that wild chickpeas limited water-loss faster than water profligate cultivated genotypes. The procedural and quantitative analyses presented through this work can be extremely beneficial for genetic analyses, prescriptive breeding, and can also be adopted for a vast number of crop types and adaptation-related traits. From a generic viewpoint, it also provides a way to overcome the bottleneck between the rate of HTP data generation and information extraction. Interested readers can find the R scripts on the open-source GitHub platform, https://github.com/KSoumya/EZTr.

## Supplementary information

**Additional file 1: Table S1.** Minimum and maximum values of Relative Humidity (%), Temperature (˚C), Vapour Pressure Deficit (kPa) and maximum of Solar Radiation (MJm^-2^) and Wind Speed (ms^-1^) measured from 20.02.2017 to 06.03.2017 at the LeasyScan HTP platform where 48 chickpea genotypes were phenotyped. **Table S2.** Estimates of the class-specific Mean Decrease in Accuracy (MDA, %) of the OOB samples from cluster 1 (C1), cluster 2 (C2) and cluster 3 (C3), the Overall Mean Decrease in Accuracy (MDA, %) and the Mean Decrease in Gini (MDG) for each feature, obtained from the Random Forest model with the least OOB error rate of 6.23%. **Table S3.** The breakpoint values of Vapour Pressure Deficit (VPD, kPa) at which maximum transpiration rate (TR) was restricted, is given for cluster 1 (C1), cluster 2 (C2), cluster 3 (C3) and at each level of environmental influence (% Split). The values shown are considering the 100% VPD level as 6.29 kPa which was the maximum value observed during the experiment. **Figure S1.** Variation in maximum TR (grams/sector/15 min) with respect to maximum VPD (kPa) for the wild (a), highTE (b) and (c) groups of genotypes. The trend lines (red dotted lines) and the equation of the linear regression between the median maximum TR and corresponding VPD values are also shown for each group. Data shown in the plots is sorted in the increasing order of maximum VPD values, as measured from 20.02.2017 – 06.03.2017. **Figure S2.** Dunn Index values plotted with respect to the increasing number of clusters from 2 to 10. **Figure S3.** The change in OOB Error Rate (%) plotted for mtry (the subset of features used for model training) values ranging from 1 to 10, during cross-validation of the unsupervised Random Forest (uRF) model

## Data Availability

The datasets used and/or analysed during the current study are available from the corresponding author on reasonable request.
