# Supplementary material for: Automated discretization of ‘transpiration restriction to increasing VPD’ features from outdoors high-throughput phenotyping data
Source: Plant Methods. 2020 Oct 16;16:140. doi: 10.1186/s13007-020-00680-8 (PMC7565372; doi:10.1186/s13007-020-00680-8)
Supplement: Supplementary file 1 — Additional file 1: Table S1. Minimum and maximum values of Relative Humidity (%), Temperature (˚C), Vapour Pressure Deficit (kPa) and maximum of Solar Radiation (MJm-2) and Wind Speed (ms-1) measured from 20.02.2017 to 06.03.2017 at the LeasyScan HTP platform where 48 chickpea genotypes were phenotyped. Table S2. Estimates of the class-specific Mean Decrease in Accuracy (MDA, %) of the OOB samples from cluster 1 (C1), cluster 2 (C2) and cluster 3 (C3), the Overall Mean Decrease in Accuracy (MDA, %) and the Mean Decrease in Gini (MDG) for each feature, obtained from the Random Forest model with the least OOB error rate of 6.23%. Table S3. The breakpoint values of Vapour Pressure Deficit (VPD, kPa) at which maximum transpiration rate (TR) was restricted, is given for cluster 1 (C1), cluster 2 (C2), cluster 3 (C3) and at each level of environmental influence (% Split). The values shown are considering the 100% VPD level as 6.29 kPa which was the maximum value observed during the experiment. Figure S1. Variation in maximum TR (grams/sector/15 min) with respect to maximum VPD (kPa) for the wild (a), highTE (b) and (c) groups of genotypes. The trend lines (red dotted lines) and the equation of the linear regression between the median maximum TR and corresponding VPD values are also shown for each group. Data shown in the plots is sorted in the increasing order of maximum VPD values, as measured from 20.02.2017 – 06.03.2017. Figure S2. Dunn Index values plotted with respect to the increasing number of clusters from 2 to 10. Figure S3. The change in OOB Error Rate (%) plotted for mtry (the subset of features used for model training) values ranging from 1 to 10, during cross-validation of the unsupervised Random Forest (uRF) model [file 13007_2020_680_MOESM1_ESM.docx]

Supplementary Table 1 Minimum and maximum values of Relative Humidity (%), Temperature (˚C), Vapour Pressure Deficit (kPa) and maximum of Solar Radiation (MJm^-2^) and Wind Speed (ms^-1^) measured from 20.02.2017 to 06.03.2017 at the LeasyScan HTP platform where 48 chickpea genotypes were phenotyped.

| **Date** | **Relative Humidity (%)** | | **Temperature (˚C)** | | **Vapour Pressure Deficit (kPa)** | | **Solar Radiation (MJm^-2^)** | **Wind Speed (ms^-1^)** |
| --- | --- | --- | --- | --- | --- | --- | --- | --- |
|  | **Min** | **Max** | **Min** | **Max** | **Min** | **Max** | **Max** | **Max** |
| 20-02-2017 | 12.02 | 68.21 | 16.70 | 39.57 | 0.60 | 6.29 | 361.80 | 3.82 |
| 21-02-2017 | 19.63 | 71.52 | 17.29 | 38.78 | 0.57 | 5.53 | 348.00 | 4.94 |
| 22-02-2017 | 18.91 | 75.85 | 18.37 | 39.29 | 0.53 | 5.64 | 335.40 | 3.61 |
| 23-02-2017 | 16.87 | 82.90 | 18.06 | 39.23 | 0.37 | 5.71 | 335.70 | 3.81 |
| 24-02-2017 | 16.26 | 70.39 | 17.95 | 38.99 | 0.62 | 5.81 | 356.70 | 3.27 |
| 25-02-2017 | 20.17 | 78.22 | 17.11 | 35.99 | 0.45 | 4.70 | 350.30 | 3.91 |
| 26-02-2017 | 15.33 | 81.48 | 15.68 | 36.16 | 0.34 | 5.08 | 367.50 | 4.56 |
| 27-02-2017 | 17.97 | 68.29 | 15.81 | 36.12 | 0.57 | 4.91 | 348.40 | 4.11 |
| 28-02-2017 | 17.03 | 74.63 | 17.62 | 36.99 | 0.52 | 5.14 | 348.90 | 4.49 |
| 01-03-2017 | 22.80 | 72.32 | 16.42 | 36.18 | 0.52 | 4.54 | 344.40 | 4.83 |
| 02-03-2017 | 19.27 | 71.79 | 17.47 | 37.03 | 0.57 | 5.07 | 347.00 | 5.26 |
| 03-03-2017 | 12.92 | 57.33 | 18.13 | 38.66 | 0.89 | 5.91 | 351.90 | 5.64 |
| 04-03-2017 | 21.46 | 68.30 | 17.39 | 38.64 | 0.74 | 5.39 | 347.90 | 3.94 |
| 05-03-2017 | 29.04 | 92.86 | 20.47 | 38.26 | 0.18 | 4.76 | 396.10 | 5.05 |
| 06-03-2017 | 37.29 | 88.76 | 20.99 | 37.76 | 0.29 | 4.05 | 350.70 | 4.61 |


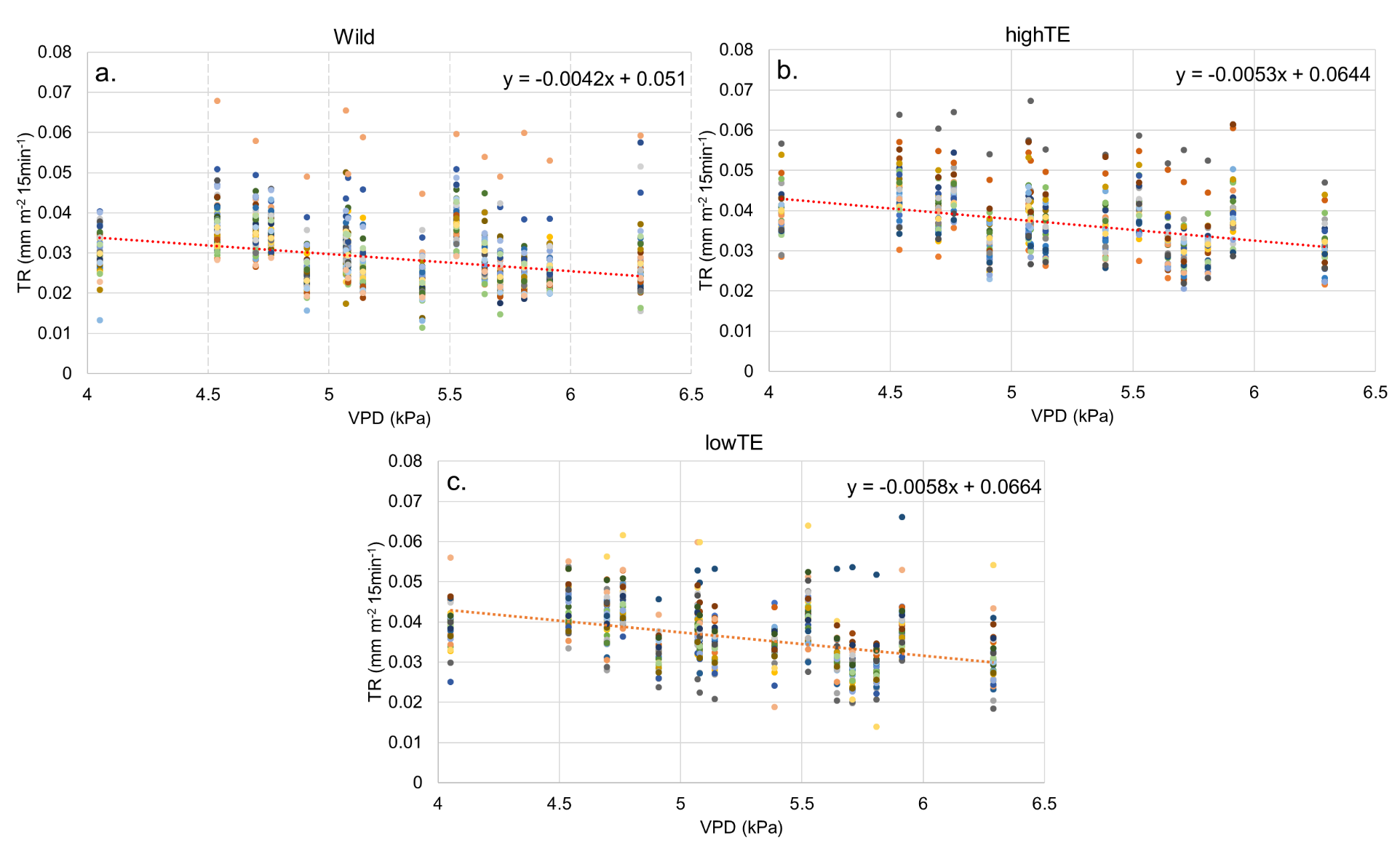


Supplementary Figure 1 Variation in maximum TR (grams/sector/15 min) with respect to maximum VPD (kPa) for the wild (a), highTE (b) and (c) groups of genotypes. The trend lines (red dotted lines) and the equation of the linear regression between the median maximum TR and corresponding VPD values are also shown for each group. Data shown in the plots is sorted in the increasing order of maximum VPD values, as measured from 20.02.2017 – 06.03.2017.

­
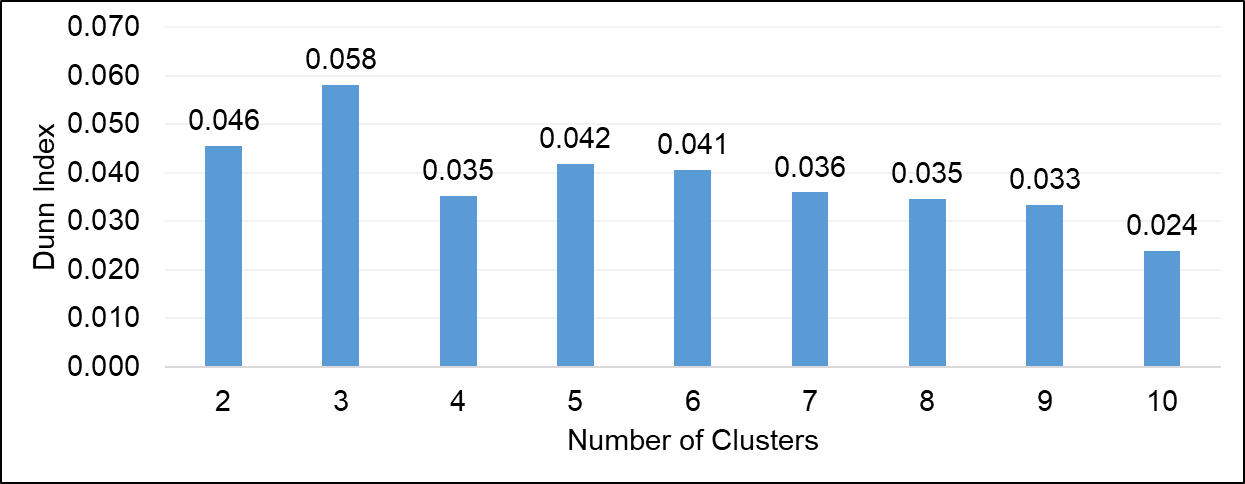


Supplementary Figure 2 Dunn Index values plotted with respect to the increasing number of clusters from 2 to 10.


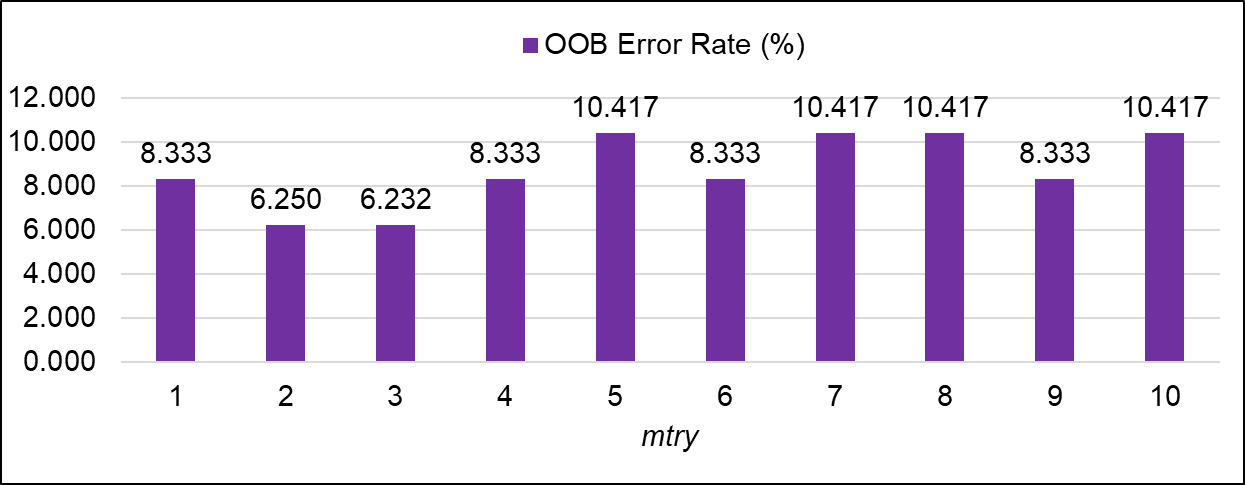


Supplementary Figure 3 The change in OOB Error Rate (%) plotted for mtry (the subset of features used for model training) values ranging from 1 to 10, during cross-validation of the unsupervised Random Forest (uRF) model

Supplementary Table 2 Estimates of the class-specific Mean Decrease in Accuracy (MDA, %) of the OOB samples from cluster 1 (C1), cluster 2 (C2) and cluster 3 (C3), the Overall Mean Decrease in Accuracy (MDA, %) and the Mean Decrease in Gini (MDG) for each feature, obtained from the Random Forest model with the least OOB error rate of 6.23%.

| Feature | Class-specific Mean Decrease in Accuracy (MDA, %) | | | Overall Mean Decrease in Accuracy (MDA, %) | Mean Decrease in Gini (MDG) |
| --- | --- | --- | --- | --- | --- |
|  | **C1** | **C2** | **C3** |  |  |
| maxTR | 14.473 | 8.923 | 12.571 | 11.721 | 5.574 |
| auc.10.15 | 10.923 | 6.229 | 7.214 | 9.043 | 4.696 |
| sd.07.19 | 7.142 | 7.858 | 11.837 | 8.037 | 4.314 |
| slope.07.maxTR | 3.040 | 5.721 | 11.323 | 6.769 | 4.199 |
| auc.07.19 | 9.083 | 4.093 | 4.552 | 6.058 | 3.246 |
| total.auc | 5.715 | 3.869 | 3.832 | 4.474 | 2.430 |
| sd.10.15 | 0.064 | 1.643 | 1.504 | 0.945 | 1.039 |
| auc.prop.10.15 | 0.140 | 0.683 | 1.542 | 0.825 | 0.947 |
| slope.maxTR.6 | 0.296 | 1.247 | 0.848 | 0.824 | 0.857 |
| curvmaxTR | 0.624 | 1.653 | -0.356 | 0.465 | 0.764 |
| slope.00.07 | -0.121 | 0.110 | 0.062 | 0.051 | 0.603 |
| slope.19.23.45 | 0.066 | 0.613 | 0.022 | 0.207 | 0.542 |
| auc.prop.night | 0.197 | 0.376 | 0.391 | 0.349 | 0.504 |
| auc.prop.07.19 | 0.250 | 0.221 | 0.143 | 0.197 | 0.472 |
| cos.sim.index | -0.100 | 0.500 | -0.338 | -0.041 | 0.390 |

Supplementary Table 3 The breakpoint values of Vapour Pressure Deficit (VPD, kPa) at which maximum transpiration rate (TR) was restricted, is given for cluster 1 (C1), cluster 2 (C2), cluster 3 (C3) and at each level of environmental influence (% Split). The values shown are considering the 100% VPD level as 6.29 kPa which was the maximum value observed during the experiment.

| **% Split** | **Breakpoint in TR response to VPD (kPa)** | | |
| --- | --- | --- | --- |
|  | **C1** | **C2** | **C3** |
| 0 | 3.52 | 5.09 | 4.97 |
| 20 | 3.71 | 4.97 | 4.91 |
| 40 | 3.84 | 4.84 | 4.84 |
| 60 | 3.90 | 4.72 | 4.84 |
| 80 | 3.77 | 4.40 | 4.78 |
| 100 | 3.90 | 4.21 | 4.97 |
